# Alexithymia and Psychological Profile in Systemic Lupus Erythematosus: Clinical and Immunological Correlates

**DOI:** 10.3390/jcm15072632

**Published:** 2026-03-30

**Authors:** Samuele Rizzo, Stefania Nicola, Richard Borrelli, Luisa Brussino, Simone Negrini

**Affiliations:** 1 Department of Internal Medicine, University of Genoa, 16132 Genoa, Italy; 2 Department of Medical Sciences, University of Turin, 10124 Turin, Italy; 3 Immunology and Allergy Unit, AO Ordine Mauriziano di Torino, 10128 Turin, Italy

**Keywords:** systemic lupus erythematosus, autoimmunity, antiphospholipid syndrome, alexithymia, TAS-20, coping strategies, quality of life, disease activity, COPE-60, psychoneuroimmunology

## Abstract

**Background/Objectives**: Systemic lupus erythematosus (SLE) is frequently accompanied by psychological distress. Alexithymia, an impairment in identifying and describing emotions, has been reported in SLE, but its clinical and serological correlates remain insufficiently characterized. We aimed to estimate the prevalence of clinically significant alexithymia in SLE and to explore its clinical, laboratory, and coping-related correlates. **Methods**: In this cross-sectional observational study, adult outpatients fulfilling the 2019 ACR/EULAR SLE classification criteria were assessed at a tertiary referral centre (2024–2025). Alexithymia was measured using the Toronto Alexithymia Scale-20 (TAS-20), and clinically significant alexithymia was defined as a total score >60. Coping strategies were assessed with the 60-item COPE inventory (Italian version). Clinical indices (SLEDAI-2K, Lupus Low Disease Activity State (LLDAS), and SLICC/ACR Damage Index (SDI)), organ involvement, antiphospholipid syndrome (APS), selected autoantibodies, complement levels, and treatments were recorded. Group comparisons and exploratory logistic regression were performed. **Results**: Sixty-eight patients were included (94.1% female). Clinically significant alexithymia was present in 23.5%. In univariate analysis, alexithymia was more frequent among patients with APS. Alexithymic participants reported higher use of emotional venting and lower use of positive reinterpretation. In an exploratory multivariable logistic regression model, APS (adjusted OR 35.79, 95% CI 3.74–341.7), emotional venting (adjusted OR 1.684, 95% CI 1.162–2.44), and positive reinterpretation (adjusted OR 0.514, 95% CI 0.349–0.755) remained associated with alexithymia. **Conclusions**: Alexithymia was frequent in this SLE cohort and, in exploratory analyses, was associated with APS and specific coping patterns. These findings suggest that assessment of emotional processing and coping may provide complementary clinical information, particularly in patients with APS, but should be interpreted as associative and hypothesis-generating.

## 1. Introduction

Systemic lupus erythematosus (SLE) is a chronic, multisystem autoimmune disorder marked by immune dysregulation, formation of autoantibodies, and widespread tissue inflammation [1]. It predominantly affects women of childbearing age and presents with a highly heterogeneous clinical course, involving a range of organ systems including the skin, joints, kidneys, cardiovascular structures, and central nervous system [2]. While substantial progress has been made in understanding the immunopathology of SLE, its neuropsychiatric and psychosomatic dimensions remain incompletely elucidated [3,4].

Psychiatric manifestations are increasingly recognized as a major component of disease burden in SLE. Neuropsychiatric SLE (NPSLE), as defined by the American College of Rheumatology (ACR), encompasses 19 distinct syndromes, five of which are purely psychiatric in nature: mood disorders, anxiety, psychosis, cognitive dysfunction, and acute confusional states [5]. The prevalence of NPSLE varies widely depending on the population studied and the diagnostic criteria applied, but some estimates suggest that up to 80% of patients with SLE experience at least one neuropsychiatric manifestation during the course of their disease [4,6]. These features not only complicate diagnosis and management but also significantly impair quality of life, treatment adherence, and long-term outcomes.

In the present study, alexithymia was examined in relation to coping strategies adopted by patients with SLE in response to disease-related and general stressors. Alexithymia is a clinically relevant but still relatively underexplored psychological construct in SLE. First described by Nemiah, Freyberger, and Sifneos in the 1970s, alexithymia is now understood as a multidimensional disturbance of emotional awareness characterised by difficulties in identifying and describing feelings and by an externally oriented thinking style [7,8,9]. While initially conceptualized as a stable personality trait, subsequent evidence suggests that alexithymia may also reflect a dynamic, state-dependent process influenced by chronic illness, pain, and emotional trauma [10]. In the context of SLE, the interaction between chronic inflammation, emotional stress, and affective dysregulation renders alexithymia a particularly relevant construct for exploration [11].

Despite its clinical relevance, the clinical and psychological correlates of alexithymia in SLE remain only partially defined. Available studies are predominantly cross-sectional, often based on small samples and focused on isolated psychological constructs. Consequently, the relationships between alexithymia, key clinical variables such as disease activity, and coping strategies have not yet been adequately characterized within a multidimensional framework. This gap in the literature supports further studies integrating psychometric assessment with detailed clinical and immunological characterization.

The present study aimed (i) to establish the prevalence of clinically significant alexithymia using validated and culturally adapted psychometric instruments; (ii) to quantify associations between alexithymia and SLE-related clinical/laboratory measures, including APS, disease activity, damage and LLDAS; (iii) to assess whether alexithymia is associated with specific coping strategies measured by the COPE inventory; and (iv) to build an exploratory multivariable model in order to identify characteristics that could help identify alexithymic patients potentially amenable to targeted intervention. 

## 2. Materials and Methods

### 2.1. Toronto Alexithymia Scale-20 (TAS-20)

The Toronto Alexithymia Scale-20 (TAS-20) is a 20-item self-report instrument widely used to assess alexithymia in clinical and research settings [12]. It includes three subscales addressing difficulty identifying feelings, difficulty describing feelings, and externally oriented thinking [13]. Items are rated on a five-point Likert scale, and the total score was used in the present study; scores >60 were considered indicative of clinically significant alexithymia. The Italian version adopted in this study has been previously validated, with satisfactory psychometric properties reported across different populations [12,13,14]. TAS-20 has also been applied in medical settings, including chronic disease populations, where alexithymia has been associated with poorer psychosocial functioning and greater emotional burden [15].

### 2.2. Coping Orientation to Problems Experienced-60 (COPE-60)

The Coping Orientation to Problems Experienced Inventory (COPE-60) is a self-report questionnaire developed to assess coping responses [16]. Consistent with the transactional model of stress and coping, it evaluates multiple coping strategies that can be grouped into broader functional domains [17]. The instrument includes 60 items organised into 15 subscales, which can be summarised into higher-order domains including social support, avoidance, positive attitude, problem orientation, and transcendence [16,18]. Items are rated on a Likert-type scale, with higher scores indicating greater use of the corresponding coping strategy [19]. The Italian adaptation (COPE-NVI) has been validated and was used in the present study [20].

### 2.3. Population and Study Design

This cross-sectional observational study enrolled adult outpatients diagnosed with SLE at a tertiary referral centre. No formal a priori sample size calculation or power analysis was performed, as the study was designed as an exploratory cross-sectional investigation. Eligibility for inclusion required a diagnosis of SLE according to the 2019 classification criteria jointly established by the American College of Rheumatology and the European League Against Rheumatism (ACR/EULAR) [21]. Participants were also required to be adults and to have the cognitive and linguistic ability to complete standardized psychological instruments. To minimize potential sources of confounding, patients with severe psychiatric or neurological comorbidities not attributable to SLE were excluded. Eligible patients were recruited consecutively between 2024 and 2025 at the outpatient clinic of our tertiary referral centre, provided that they agreed to participate and gave informed consent.

All participants provided informed consent after receiving full information about the study. The study was conducted in accordance with the Declaration of Helsinki and was approved by the local Ethics Committee (Comitato Etico Territoriale interaziendale AOU Città della Salute e della Scienza di Torino; protocol code #0116953; date of approval: 9 October 2023). At the time of psychological assessment, all patients underwent a comprehensive clinical and laboratory evaluation. Laboratory investigations included a complete blood count, serum protein electrophoresis, liver and renal function tests, and urinalysis. In cases of renal involvement, 24-h proteinuria was quantified. Complement levels (C3 and C4) were assessed, and autoimmune serology included anti-double stranded DNA antibodies, anticardiolipin, anti-β2 glycoprotein I antibodies, and lupus anticoagulant testing. For laboratory variables not routinely assessed at the study visit, results were treated as historical (ever recorded) and are reported separately.

Disease activity was measured using the Systemic Lupus Erythematosus Disease Activity Index 2000 (SLEDAI-2K) [22], while the Lupus Low Disease Activity State (LLDAS) [23] criteria were applied to identify patients in a stable, minimally active phase. Cumulative organ damage was quantified using the Systemic Lupus International Collaborating Clinics/American College of Rheumatology Damage Index (SLICC/ACR DI) [24]. Antiphospholipid syndrome was defined according to the 2023 ACR/EULAR classification criteria [25], while neuropsychiatric involvement was attributed according to the 1999 ACR case definitions [5].

Psychological functioning was evaluated through two self-administered, validated instruments. The Italian version of the twenty-item Toronto Alexithymia Scale (TAS-20) was used to assess alexithymia. This scale measures three core components: difficulty identifying feelings, difficulty describing feelings, and externally oriented thinking. Although subscores can be calculated, the total score was used in this study, with a cut-off of >60 indicating clinically significant alexithymia.

Coping strategies were assessed using the Italian version of the sixty-item Coping Orientation to Problems Experienced Inventory (COPE-60). This multidimensional instrument measures fifteen specific coping strategies, organised into five overarching domains: social support (e.g., emotional and instrumental support, venting), avoidance (e.g., denial, disengagement, substance use), positive attitude (e.g., acceptance, positive reinterpretation), problem orientation (e.g., planning, active coping), and transcendence (e.g., humour, turning to religion). Each item is rated on a Likert scale, and higher scores indicate greater use of a given coping strategy.

### 2.4. Statistical Analysis

All statistical analyses were performed using Jamovi v2.7 [26,27,28,29]. No control group was included, as this was an exploratory study. Descriptive statistics are reported as *n* (%) for categorical variables. Continuous variables are summarized as median (interquartile range) unless normality assumptions were met. Group comparisons between participants with and without alexithymia were conducted using Fisher’s exact test for categorical variables and the Mann–Whitney U test for non-normally distributed continuous variables; Student’s *t* test was used only when distributional assumptions were satisfied. For univariate analyses, effect estimates are reported alongside *p* values (e.g., odds ratios for categorical predictors and nonparametric effect sizes for questionnaire subscales). Given the exploratory evaluation of 15 COPE subscales and the absence of formal correction for multiple testing, these nominal associations should be interpreted cautiously and regarded as hypothesis-generating rather than confirmatory. An exploratory multivariable logistic regression model was used to evaluate factors associated with alexithymia. Model parsimony was prioritized because of the limited number of events, and the resulting estimates were therefore regarded as exploratory. In the final multivariable model, 16 alexithymia events were available for 3 retained predictors, corresponding to approximately 5.3 events per variable; this is below conventional recommendations for stable multivariable logistic regression estimates and reinforces the exploratory nature of the analysis. Collinearity among candidate predictors was assessed prior to model fitting, including through the variance inflation factor (VIF). No missing data were present for the variables included in the regression model; therefore, the analysis was performed on complete cases and no imputation was required. A formal assessment of linearity in the logit for continuous predictors was not pre-specified. Results are presented as adjusted odds ratios (ORs) with 95% confidence intervals (CIs). Classification-based metrics (accuracy, sensitivity, specificity, and AUC) are reported as descriptive measures.

## 3. Results

Sixty-eight patients were included (94.1% female). At the time of evaluation, the median SLEDAI-2K score was 4 (IQR 2–6), 41.2% met criteria for LLDAS, and the median SDI was 1 (IQR 0–2). Clinically significant alexithymia (TAS-20 > 60) was present in 23.5% of the sample. Clinical and laboratory features are summarized in Table 1 and Table 2.

In univariate analysis, alexithymia was significantly more frequent among patients with APS (6/11, 54.5%) than among those without APS (10/57, 17.5%; OR 5.64, *p* = 0.013), but no association was found with antiphospholipid antibody positivity alone or with other clinical manifestations or laboratory parameters. A higher cumulative damage score assessed with the SDI was noted in alexithymic patients (Mann–Whitney U test, *p* = 0.034), although this was not confirmed in subsequent analysis, while no difference in disease activity was observed. A higher degree of denial coping was observed among patients who were not in a low disease activity state (LLDAS) (Mann–Whitney U test, *p* = 0.013).

Higher TAS-20 scores were found in patients with APS (Student’s *t* test, *p* = 0.043) and in patients with SLE and ever recorded neuropsychiatric manifestations (Student’s *t* test, *p* = 0.043), although the latter was not confirmed by subsequent analysis.

Among coping domains (Table 3), alexithymic participants reported higher use of ‘focus on and venting of emotions’ (*p* = 0.014) and lower use of ‘positive reinterpretation’ (*p* < 0.001). No significant differences were observed in the remaining COPE subscales (Table 4). Given the exploratory evaluation of 15 COPE subscales, these nominal associations should be interpreted cautiously.

The table shows the descriptive statistics of the COPE-60 results with the related median and interquartile range (IQR) for the non-parametric variables and mean and standard deviation (SD) for the parametric one, based on the Shapiro–Wilk test.

In an exploratory multivariable logistic regression model (Table 5), APS showed the strongest association with alexithymia (adjusted OR 35.79, 95% CI 3.74–341.7; *p* = 0.002). Higher emotional venting was associated with higher odds of alexithymia (adjusted OR 1.684 per 1-point increase, 95% CI 1.162–2.44; *p* = 0.006), whereas positive reinterpretation was associated with lower odds (adjusted OR 0.514 per 1-point increase, 95% CI 0.349–0.755; *p* < 0.001). Given the limited number of events and the wide confidence interval observed for APS, these estimates should be interpreted as exploratory and potentially unstable. Neuropsychiatric manifestations and SDI were not retained because they did not improve the exploratory model. McFadden’s R^2^ for the exploratory model was 0.477 and is reported as a descriptive fit index (Table 6).

Estimates are reported as odds ratios (ORs) with 95% confidence intervals for TAS-20 > 60 vs. TAS-20 ≤ 60. APS: antiphospholipid syndrome; NPSLE: neuropsychiatric systemic lupus erythematosus; SDI: SLICC/ACR Damage Index. The exploratory multivariable binomial logistic regression model retained APS, venting of emotions, and lower positive reinterpretation as variables associated with alexithymia; given the limited number of events, these estimates should be interpreted cautiously.

McFadden’s R^2^ (0.477) is reported as a descriptive fit index for the exploratory model. The prediction model showed an apparent accuracy of 88.2%, sensitivity of 68.8%, specificity of 94.2%, and an AUC of 0.918. These are in-sample estimates and should not be interpreted as evidence of clinical screening utility without validation.

## 4. Discussion

Psychological functioning and immune regulation are increasingly recognized as interacting dimensions in systemic autoimmune diseases, including SLE [30,31]. In this context, the present study characterized the psychological profile of a clinically stable SLE outpatient cohort. The demographic and disease-related features of the sample were consistent with those reported in tertiary-care SLE cohorts, in which patients are predominantly female, middle-aged, and frequently in a low disease activity state, thereby reducing the potential influence of active inflammation on psychological assessment [2,32].

Alexithymia is not merely “a difficulty in expressing feelings” in colloquial terms; rather, it reflects a multidimensional disturbance in affective cognition. It involves impairments in the capacity to recognise discrete emotional states within oneself, to discriminate them from bodily sensations of arousal, and to translate these subjective states into language accessible for interpersonal communication [9]. This deficit often coexists with a cognitive style that privileges external, concrete, and operational aspects of experience over introspection and symbolic elaboration [33]. In chronic illness, such impairments may hinder adaptive emotional regulation, impede the formation of accurate illness appraisals, and exacerbate stress reactivity, thereby influencing both mental health and disease course through psychoneuroimmunological pathways [9,33].

Existing literature suggests that the prevalence of alexithymia in patients with SLE is significantly higher than in the general population, where it is estimated at around 10%. Reported rates in SLE cohorts vary from 17.5% to 55.9%, with differences likely attributable to sample characteristics, disease severity, and the psychological instruments employed [11,15]. Alexithymia has also been associated with psychological distress, pain-related burden, and poorer psychosocial functioning, all of which may contribute to overall disease burden [34,35]. Alexithymia, defined as a multidimensional impairment in identifying, describing, and processing one’s own emotional states, was present in 23.5% of participants. This prevalence aligns with earlier findings suggesting increased rates of alexithymia among individuals with SLE compared to the general population [11,15]. However, in the absence of a contemporaneous healthy control group, this comparison remains indirect and based on previously published estimates rather than on within-study controls. In the exploratory multivariable model, APS showed the strongest association with alexithymia. Alexithymia was observed in 6 of 11 patients with APS (54.5%) compared with 10 of 57 patients without APS (17.5%), suggesting that the APS subgroup contributed substantially to the overall signal observed in this cohort. In contrast, seropositivity for antiphospholipid antibodies in the absence of clinically defined APS was not associated with alexithymic features. These findings underscore the importance of distinguishing between isolated antiphospholipid antibody positivity and overt APS, which is defined by the coexistence of persistent aPL and clinical manifestations, when considering psychological correlates [25,36]. No association was observed between alexithymia and SLE disease activity or achievement of LLDAS, although alexithymic patients showed greater accumulated organ damage as measured by the SLICC/ACR Damage Index. This is consistent with previous evidence suggesting that, at least in a subgroup of patients with SLE, alexithymia may not simply reflect current disease activity but may represent a more stable vulnerability [11,15,33].

While neuroimaging was not conducted in this study, prior diffusion tensor imaging work has demonstrated structural correlates of alexithymia, including microstructural alterations in white matter tracts involved in emotional processing and regulation [37]. Our findings suggest an association between APS and alexithymia in this cohort; however, any neurocognitive or vascular-mediated interpretation remains speculative, as no neuroimaging or formal cognitive assessment was performed and the study design was cross-sectional. This interpretation is supported by APS studies showing cognitive deficits, particularly in complex attention and verbal fluency, and an association between cognitive dysfunction and white matter lesions on brain MRI [38]. A later systematic review similarly linked cognitive dysfunction in APS/aPL-positive cohorts with white matter hyperintensities, ischaemic lesions, and cortical atrophy, while also emphasizing the heterogeneity of the evidence base [39].

Coping refers to the cognitive and behavioural efforts through which individuals attempt to manage demands appraised as taxing or exceeding their available resources [17]. Although coping strategies are not inherently adaptive or maladaptive, their functional significance depends on the context, the controllability of stressors, and the flexibility with which they are used. In chronic disease settings, coping may influence psychological outcomes such as mood, anxiety, and quality of life, as well as illness-related behaviours including treatment adherence, use of social support, and self-management [18,19]. In this context, persistent avoidance, behavioural disengagement, or poorly regulated emotional discharge may contribute to distress and functional impairment, whereas planning, acceptance, and cognitive reappraisal are more often associated with better adaptation to disease-related burden [18,19].

In our cohort, most patients employed adaptive strategies, particularly planning, acceptance, and positive reinterpretation. Maladaptive strategies such as substance use were infrequent. Denial coping was more frequently used by patients who were not in a low disease activity state (LLDAS). However, among alexithymic individuals, a distinctive coping pattern emerged, marked by increased use of emotional venting and diminished use of positive reinterpretation. The paradoxical coexistence of high emotional discharge with low introspective ability may reflect efforts to regulate affective arousal in the absence of internal emotional clarity. This pattern is also clinically meaningful because, in SLE, positive reinterpretation has been associated with better health-related quality of life, whereas focus on and venting of emotion has been associated with poorer health-related quality of life [40].

In the exploratory multivariable model, in addition to APS, increased reliance on emotional venting was associated with higher odds of alexithymia, whereas positive reinterpretation was associated with lower odds. These findings suggest that specific coping dimensions may be relevant correlates of alexithymia in SLE. However, the large adjusted OR observed for APS and its wide confidence interval indicate substantial uncertainty around the effect estimate, likely related to the limited number of alexithymia events and the risk of overfitting. The apparent protective association of positive reinterpretation is consistent with prior SLE evidence linking this coping strategy to better health-related quality of life [40]. Although the combination of APS and selected coping subscales showed promising discrimination in this dataset, any performance estimates represent apparent (in-sample) results and should not be interpreted as evidence of clinical screening utility without internal or external validation. Given the modest sample size and limited number of events, these findings should be considered hypothesis-generating, and the magnitude of the multivariable estimates should be interpreted cautiously pending validation in independent cohorts.

A psychoneuroimmunological perspective may help contextualize the role of alexithymia in autoimmune diseases such as SLE. This framework emphasizes the bidirectional interplay between psychological processes, neuroendocrine pathways, and immune responses [30]. Within this model, emotional stress and affective dysregulation may alter homeostatic mechanisms through activation of the hypothalamic–pituitary–adrenal axis and the sympathetic-adrenal-medullary system, with downstream effects on immune cell function, cytokine release, and inflammatory signalling [30]. Conversely, systemic inflammation may influence central nervous system functioning through cytokine-related mechanisms, neurotransmitter alterations, and microvascular injury [31]. Overall, this bidirectional interaction supports the clinical relevance of addressing emotional health within comprehensive SLE care.

Taken together, these findings support the view that emotional dysregulation may represent a clinically relevant dimension of disease burden in SLE. Psychological and biological factors may be interrelated in shaping symptoms, coping patterns, and functional impairment, although their temporal and causal relationships cannot be determined in the present study. In practical terms, a structured assessment of emotional processing and coping may be particularly informative in patients with APS or in those who report disproportionate distress despite relatively low disease activity. A recent SLE-focused review similarly suggested that alexithymia and trauma-related psychopathology may contribute to disease burden through altered stress processing, hypothalamic–pituitary–adrenal axis dysfunction, pain amplification, and immune dysregulation [41].

### Limitations

Although this study employed rigorous methods and a well-characterised sample, several limitations must be acknowledged. The cross-sectional design precludes causal inference. The single-centre setting and the relatively stable clinical profile of the cohort, with 41.2% of patients meeting LLDAS at the time of assessment, may limit generalizability to patients with more active or severe SLE. No contemporary healthy control group was included; accordingly, the observed alexithymia prevalence should be interpreted as cohort-specific rather than as a definitive estimate of SLE-specific prevalence. Comparisons with general-population estimates are therefore indirect and literature-based, and the inclusion of a contemporaneous control group would have strengthened the interpretation of prevalence findings. The modest sample size, particularly the number of alexithymia cases, increases uncertainty around multivariable estimates, limits statistical power, and may inflate apparent model performance. In the final exploratory regression model, only 16 alexithymia events were available for 3 retained predictors (approximately 5.3 events per variable), increasing the risk of overfitting and unstable effect estimates. In addition, because the regression model was exploratory and based on a limited number of events, a formal assessment of linearity in the logit for continuous predictors was not pre-specified. The association with APS should also be interpreted in light of the small APS subgroup, which contributed a substantial proportion of alexithymic cases. In addition, no neuroimaging or formal cognitive assessment was performed in this cohort; therefore, any neurovascular or neurocognitive interpretation of the APS–alexithymia association remains speculative. The study relied on self-report questionnaires, which may be influenced by reporting bias. In addition, analyses across individual COPE subscales were exploratory and were not adjusted for multiple testing; accordingly, nominal *p* values should be interpreted cautiously, and the observed coping associations should be regarded as hypothesis-generating rather than confirmatory. Future studies should employ pre-specified multivariable models, perform internal validation (e.g., bootstrap), and aim for external validation in independent cohorts. Longitudinal designs could clarify temporal relationships between APS, coping patterns, and alexithymia. Randomised trials investigating psychotherapeutic or cognitive interventions tailored to alexithymic profiles may also clarify whether such traits are modifiable and whether such modification can improve patient outcomes. In addition, depression, anxiety, and fatigue were not formally measured in the present study. This is relevant because prior SLE work has shown that depression is closely linked to alexithymia and may partly account for its apparent prevalence and correlates [35]. The absence of these additional variables limits our ability to determine whether the observed associations were independent of broader mood-related or symptom-burden dimensions. Future studies should therefore incorporate validated measures of depression, anxiety, and fatigue within pre-specified multivariable models.

## 5. Conclusions

Alexithymia was common in this SLE outpatient cohort and, in exploratory analyses, was associated with APS and a specific coping pattern characterised by greater emotional venting and lower positive reinterpretation. These findings suggest that assessment of emotional processing and coping styles may offer complementary clinical information alongside standard clinical and laboratory evaluation, particularly in patients with APS, although the observed associations should be interpreted cautiously. Given the cross-sectional design, the relatively stable single-centre cohort, and the modest sample size, results should be interpreted as associative and hypothesis-generating; longitudinal studies and validated multivariable models are needed to clarify temporal relationships and clinical utility.

## Figures and Tables

**Table 1:** Disease manifestations ever recorded and active within 30 days before psychological assessment.

Disease Manifestations	Ever Recorded *n* (%)	Ongoing 30 Days *n* (%)
Articular	56 (82.4)	5 (7.4)
Hematologic	45 (66.2)	6 (8.8)
Leukopenia	33 (48.5)	4 (5.9)
Thrombocytopenia	23 (33.8)	2 (2.9)
Haemolytic anaemia	20 (29.4)	-
Mucocutaneous	39 (57.4)	1 (1.5)
Renal	38 (55.9)	8 (11.8)
Malar rash	29 (42.6)	1 (1.5)
Photosensitivity	20 (29.4)	-
Serositis	20 (29.4)	1 (1.5)
Neuropsychiatric	18 (26.5)	1 (1.5)
DLE	10 (14.7)	-
SCLE	2 (2.9)	-

DLE: discoid lupus erythematosus; SCLE: subacute cutaneous lupus erythematosus. The table shows the clinical manifestations in patient history (ever recorded) or active manifestations during the last 30 days assessed at the control visit.

**Table 2:** Autoantibody and laboratory features (ever recorded and at the psychological assessment).

Laboratory Features	Ever Recorded *n* (%)	Time of Evaluation *n* (%)
anti-dsDNA Ab	45 (66.2)	24 (35.3)
anti-Ro/SSA Ab	31 (45.6)	Not routinely repeated
anti-La/SSB Ab	11 (16.2)	Not routinely repeated
anti-Sm Ab	16 (23.5)	Not routinely repeated
Complement consumption *	55 (80.9)	30 (44.1)
APL **	27 (39.7)	27 (39.7)
APS	11 (16.2)	11 (16.2)

APL: antiphospholipid antibodies; APS: antiphospholipid syndrome. Overall values refer to historical (ever recorded) positivity abstracted from medical records; these assays were not routinely repeated at the study visit. Time-of-evaluation values refer to the positivity repeated/confirmed at the study visit. * Complement consumption defined as low C3 and/or C4 below the local laboratory reference range. ** aPL positivity refers to anticardiolipin and/or anti-β2 glycoprotein I antibodies and/or lupus anticoagulant positivity assessed at the study visit. Percentages are calculated using *n* tested at visit as denominator.

**Table 3:** Description of the COPE-60 results.

COPE-60 Domain	Mean/Median	SD/IQR	Shapiro–Wilk W	*p*
Positive reinterpretation	12.00	4.00	0.949	0.008
Mental disengagement	9.00	4.00	0.956	0.017
Focus on and venting emotions	9.00	4.00	0.959	0.027
Seeking of instrumental social support	11.00	5.00	0.947	0.006
Active coping	12.00	3.00	0.956	0.017
Denial	7.00	4.00	0.925	<0.001
Turning to religion	8.00	7.25	0.854	<0.001
Humour	8.00	4.00	0.918	<0.001
Behavioural disengagement	8.00	3.25	0.960	0.028
Restraint coping	10.00	2.25	0.963	0.041
Seeking of emotional social support	9.50	6.00	0.954	0.013
Alcohol or drug use	4.00	0.00	0.420	<0.001
Acceptance	11.00	4.00	0.558	<0.001
Suppression of competing activities	10.18	2.60	0.983	0.499
Planning	11.00	3.25	0.964	0.048

**Table 4:** Univariate analysis for COPE-60 domains and alexithymic patients.

COPE-60 Domain	Test	Statistic	*p*	Effect Size
Positive reinterpretation	Mann–Whitney U	181	<0.001	0.6322
Mental disengagement	Mann–Whitney U	308	0.060	−0.3005
Focus on and venting emotions	Mann–Whitney U	266	0.014	−0.4063
Seeking of instrumental social support	Mann–Whitney U	407	0.624	0.1238
Active coping	Mann–Whitney U	323	0.095	0.3101
Denial	Mann–Whitney U	355	0.224	−0.2380
Turning to religion	Mann–Whitney U	431	0.876	−0.0276
Humour	Mann–Whitney U	398	0.537	0.1478
Behavioural disengagement	Mann–Whitney U	335	0.133	−0.2163
Restraint coping	Mann–Whitney U	396	0.518	−0.1382
Seeking of emotional social support	Mann–Whitney U	440	0.983	0.0361
Alcohol or drug use	Mann–Whitney U	411	0.527	−0.0865
Acceptance	Mann–Whitney U	403	0.589	0.1310
Suppression of competing activities	Student’s *t*	−0.643	0.523	−0.1825
Planning	Mann–Whitney U	342	0.283	0.1779

Note. The Mann–Whitney U test was used for non-parametric data, while Student’s *t* test was used for parametric data. Effect size is reported as rank-biserial correlation for non-parametric variables and Cohen’s d for parametric variables to quantify the magnitude of group differences. Positive reinterpretation was negatively associated with the presence of alexithymia defined as a TAS-20 score >60, while focus on and venting of emotions was positively associated. Because the 15 COPE subscales were explored without formal correction for multiple testing, these *p* values should be interpreted cautiously and should not be considered confirmatory.

**Table 5:** Univariate and multivariable logistic regression models for alexithymia.

Variable	Univariate OR	Univariate 95% CI	Univariate *p*	Multivariable OR	Multivariable 95% CI	Multivariable *p*
APS	5.64	1.43–22.18	0.013	35.79	3.74–341.7	0.002
NPSLE	2.9	0.99–9.54	0.08			
SDI	1.391	0.996–1.942	0.053			
Positive reinterpretation	0.643	0.497–0.830	<0.001	0.514	0.349–0.755	<0.001
Focus on and venting of emotions	1.323	1.046–1.673	0.019	1.684	1.162–2.44	0.006

**Table 6:** Model fit measures.

Model	Deviance	AIC	McFadden’s R^2^	χ^2^	df/*p*
1	38.8	46.8	0.477	35.4	3/<0.001

## Data Availability

The datasets generated and analysed during the current study are not publicly available due to privacy reasons but are available from the corresponding author upon reasonable request.
